# Supplementary material for: Immune–Pathological Correlates of Disease Severity in New-World Kala-Azar: The Role of Parasite Load and Cytokine Profiles
Source: Pathogens. 2025 Jun 20;14(7):615. doi: 10.3390/pathogens14070615 (PMC12299585; doi:10.3390/pathogens14070615)
Supplement: Supplementary file 1 [file pathogens-14-00615-s001.zip › Supplemental figure.pdf]

```
      Source |       SS           df       MS      Number of obs   =        67
-------------+----------------------------------   F(6, 60)        =      2.50
       Model |  117.471559         6  19.5785932   Prob > F        =    0.0315
    Residual |  469.194716        60  7.81991193   R-squared       =    0.2002
-------------+----------------------------------   Adj R-squared   =    0.1203
       Total |  586.666275        66  8.88888295   Root MSE        =    2.7964

------------------------------------------------------------------------------
     lnkdnap |      Coef.   Std. Err.      t    P>|t|     [95% Conf. Interval]
-------------+----------------------------------------------------------------
         il1 |   .1333955   .1391706     0.96   0.342    -.1449871    .4117781
         il6 |   .0007843   .0027095     0.29   0.773    -.0046356    .0062042
        il10 |   .0131416   .0089001     1.48   0.145    -.0046613    .0309444
        il12 |  -.5212569   .2334427    -2.23   0.029    -.9882118   -.054302
         tnf |  -.0179996   .1736329    -0.10   0.918    -.3653172     .329318
         tgf |   .0096413   .0077135     1.25   0.216     -.005788    .0250705
       _cons |   6.048545   .5614512    10.77   0.000     4.925476    7.171615
------------------------------------------------------------------------------
```

```
Iteration  1:  WLS sum of weighted deviations =   70.22267

Iteration  1: sum of abs. weighted deviations =    70.3925
Iteration  2: sum of abs. weighted deviations =  70.224981
Iteration  3: sum of abs. weighted deviations =  69.962491
Iteration  4: sum of abs. weighted deviations =   69.63792
Iteration  5: sum of abs. weighted deviations =  69.483973
Iteration  6: sum of abs. weighted deviations =  69.439905

Median regression                                   Number of obs =         67
  Raw sum of deviations 76.24166 (about 6.9122553)
  Min sum of deviations  69.4399                    Pseudo R2     =     0.0892

------------------------------------------------------------------------------
     lnkdnap |      Coef.   Std. Err.      t    P>|t|     [95% Conf. Interval]
-------------+----------------------------------------------------------------
         il1 |   .0930416   .1856988     0.50   0.618    -.2785408     .464624
         il6 |  -.0000258   .0040323    -0.01   0.995    -.0080943    .0080428
         il8 |  -.0011936   .0019178    -0.62   0.536    -.0050311    .0026439
        il10 |   .0099914   .0118901     0.84   0.404    -.0138008    .0337835
        il12 |  -.5488185   .3092857    -1.77   0.081    -1.167698    .0700607
         tnf |   .0911563   .2303905     0.40   0.694     -.369854    .5521667
         tgf |   .0049508   .0103891     0.48   0.635    -.0158377    .0257393
       _cons |    6.76311   .7608958     8.89   0.000     5.240561    8.285659
------------------------------------------------------------------------------
```

Supplemental Figure S1. Above: output of the multivariate linear regression analysis for prediction of of plasma parasite kDNA load by plasma cytokines. Bellow: output of the multivariate quantile regression analysis for prediction of plasma parasite kDNA load by plasma cytokines.
