# Supplementary material for: Immune–Pathological Correlates of Disease Severity in New-World Kala-Azar: The Role of Parasite Load and Cytokine Profiles
Source: Pathogens. 2025 Jun 20;14(7):615. doi: 10.3390/pathogens14070615 (PMC12299585; doi:10.3390/pathogens14070615)
Supplement: Supplementary file 1 [file pathogens-14-00615-s001.zip › supplemental table corr indicadores de gravidade.pdf]

Supplementary material

Supplementary Table S1. Pearson's correlation coefficient matrix between variables used to estimate the severity of kala-azar.

Variable	Death Pearson's ρ (*p*-value)	Chance of death > 10% Pearson's ρ (*p*-value)	Reported bleeding Pearson's ρ (*p*-value)	Detected bleeding Pearson's ρ (*p*-value)	Sepsis syndrome Pearson's ρ (*p*-value)	Any bacterial of infection Pearson's ρ (*p*-value)
Death	-					
Chance of death > 10%	**0.33 (0.0043)**	-				
Reported bleeding	**0.47 (0.0000)**	0.21 (0.0838)	-			
Detected bleeding	0.17 (0.1434)	**0.34 (0.0031)**	**0.47 (0.0000)**	-		
Sepsis syndrome	**0.43 (0.0002)**	0.13 (0.2975)	**0.32 (0.0070)**	0.10 (0.3852)	-	
Any bacterial infection	0.22 (0.0586)	0.13 (0.2916)	0.22 (0.0586)	0.16 (0.1740)	**0.59 (0.0000)**	-
